# Adverse Events Following Pandemic A (H1N1) 2009 Monovalent Vaccines in Pregnant Women — Taiwan, November 2009–August 2010

**DOI:** 10.1371/journal.pone.0023049

**Published:** 2011-08-05

**Authors:** Wan-Ting Huang, Wan-Chin Chen, Hwa-Jen Teng, Wei-I Huang, Yu-Wen Huang, Chien-Wen Hsu, Jen-Hsiang Chuang

**Affiliations:** 1 Taiwan Centers for Disease Control, Taipei, Taiwan; 2 Taiwan Drug Relief Foundation, Taipei, Taiwan; 3 Taiwan Food and Drug Administration, Taipei, Taiwan; 4 College of Bioresources and Agriculture, Institute of Biotechnology, National Taiwan University, Taipei, Taiwan; University of Witwatersrand, South Africa

## Abstract

**Background:**

During the 2009 H1N1 pandemic, pregnant women were prioritized to receive the unadjuvanted or MF59®-adjuvanted pandemic A (H1N1) 2009 monovalent vaccines (“2009 H1N1 vaccines”) in Taiwan regardless of stage of pregnancy. Monitoring adverse events following 2009 H1N1 vaccination in pregnant women was a priority for the mass immunization campaign beginning November 2009.

**Methods/Findings:**

We characterized reports to the national passive surveillance from November 2009 through August 2010 involving adverse events following 2009 H1N1 vaccines among pregnant women. Reports from the passive surveillance were matched to a large-linked database on a unique identifier, date of vaccination, and date of diagnosis in a capture-recapture analysis to estimate the true number of spontaneous abortion after 2009 H1N1 vaccination. We verified 16 spontaneous abortions, 11 stillbirths, 4 neonatal deaths, 4 nonpregnancy-specific adverse events, and 2 inadvertent immunizations in recipients who were unaware of pregnancy at time of vaccination. The Chapman capture-recapture estimator of true number of spontaneous abortion after 2009 H1N1 vaccination was 329 (95% confidence interval [CI] 196–553). Of the 14,474 pregnant women who received the 2009 H1N1 vaccines, the estimated risk of spontaneous abortion was 2.3 (95% CI, 1.4–3.8) per 100 pregnancies, compared with a local background rate of 12.8 (95% CI, 12.8–12.9) per 100 pregnancies.

**Conclusions:**

The passive surveillance provided rapid initial assessment of adverse events after 2009 H1N1 vaccination among pregnant women. Its findings were reassuring for the safety of 2009 H1N1 vaccines in pregnancy.

## Introduction

Pregnant women are at higher risk for complications and death from pandemic A (H1N1) 2009 (“2009 H1N1”) infection [1]–[3]. This increased risk of morbidity and mortality has been observed in two previous influenza pandemics (1918–19 and 1957–58) and with seasonal influenza [4]–[6]. In Taiwan, pregnant women infected with 2009 H1N1 were 2.7 times more likely to be hospitalized than nonpregnant women of reproductive age, and 50% of the hospitalized required intensive care [7]. On November 1, 2009, the Taiwan government began a nationwide 2009 H1N1 vaccination program using two types of 2009 H1N1 vaccines: an inactivated vaccine without adjuvant (Adimmune Corporation, Taichung, Taiwan) and an MF59®-adjuvanted vaccine (Novartis Vaccines and Diagnostics, Sovicille, Italy). The Advisory Committee on Immunization Practices in Taiwan recommended and prioritized pregnant women to receive either the unadjuvanted or the MF59®-adjuvanted 2009 H1N1 vaccines, regardless of stage of pregnancy.

Because the manufacturing process for 2009 H1N1 vaccine without adjuvant was the same as those used for seasonal influenza vaccine, the safety profile to pregnant women and their infants was expected to be the same as the seasonal vaccine product, which has an excellent safety record [6]. Information about the safety of MF59®-adjuvanted influenza vaccine in pregnancy, however, was limited as pregnant women are classically excluded from participation in clinical trials of new vaccines [8]. Beginning November 2009, a postlicensure safety surveillance strategy has been implemented in Taiwan to rapidly identify and evaluate new or unusual adverse events among 2009 H1N1 vaccine recipients [9]. We report on the adverse event profile following 2009 H1N1 vaccines in pregnant women.

## Materials and Methods

### Data collection

Taiwan Centers for Disease Control (TCDC) and Taiwan Food and Drug Administration collaboratively established a national passive surveillance system for adverse event following immunization (AEFI) in concert with the 2009 H1N1 vaccination program [9]. Patients or their parents, healthcare providers, manufacturers, and others were encouraged to report any health event that occurs in patients after receipt of 2009 H1N1 vaccines at any time interval to the system, regardless of causality. Reports were categorized as serious if the adverse event involved death, life-threatening illness, hospitalization, prolongation of hospitalization, permanent disability, or congenital anomaly [10]. Medical records were sought for reports coded as serious, reports suggestive of adverse events of special interest (AESIs) [11], and reports involving pregnancy-specific adverse events.

We searched the passive surveillance database for reports received from November 1, 2009 through August 31, 2010 on pregnant women vaccinated with the 2009 H1N1 vaccines. Each report was investigated by a standard protocol at time of report received. The protocol requested blood and tissue specimens, which were subject for further relevant diagnostic tests, placental or umbilical cord pathology, or fetal autopsy (Table S1). A TCDC physician (WTH) prospectively reviewed all reports, medical records, and results on the laboratory or pathologic investigations. Adverse events were classified by the timing of exposure to 2009 H1N1 vaccines during the first (0–13 weeks), second (14–27 weeks) and third (≥28 weeks) trimester of pregnancy, and by the outcomes including pregnancy-specific and nonpregnancy-specific adverse events. We defined spontaneous abortion as natural loss of conceptuses at <20^th^ week of pregnancy and stillbirth as nonviable conceptuses at ≥20^th^ week of gestation.

Data collection was conducted as part of a public health response to the 2009 H1N1 pandemic and therefore did not require approval by an institutional review board or informed consent.

### Data matching and capture-recapture analysis

In addition to the passive surveillance, TCDC developed a nationwide large-linked database (LLDB) of computerized vaccinations and medical records for 2009 H1N1 vaccine safety hypothesis testing [9]. The *International Classification of Diseases*, *Ninth Revision*, *Clinical Modification* (ICD-9-CM) diagnoses of spontaneous abortion (codes 631, 632, 634*, 637*, 761.8) were prospectively collected from the daily updated National Health Insurance (NHI) database. This LLDB had recorded 62% of the 5.6 million doses of 2009 H1N1 vaccines administered to the Taiwan population from November 1, 2009 through August 31, 2010 (TCDC, unpublished data).

Because the proportion of the vaccinated population that experiences an AEFI cannot be directly estimated with the passive surveillance data [12], we used the capture-recapture methodology to assess the true numbers of spontaneous abortion after 2009 H1N1 vaccination for the unadjuvanted and MF59®-adjuvanted vaccines [13]. We matched all reports of spontaneous abortion from the passive AEFI surveillance to cases after 2009 H1N1 vaccination that occurred between November 1, 2009 and August 31, 2010 from the LLDB on a unique identifier assigned to each resident, date of vaccination, and date of diagnosis. The reported dates of vaccination and diagnosis in the two data sources were allowed to differ by up to 7 days to account for recall bias in the passive surveillance reports. We calculated Chapman estimators of the true number of spontaneous abortion after 2009 H1N1 vaccination as *N = [(b+1)(c+1)/(a+1)]−1*, in which *a* is the number of cases captured in both sources, *b* is the number of cases captured in the passive surveillance database, and *c* is the number of cases captured in the LLDB [14]. Variances for Chapman estimators were calculated using the formula derived by Serber as *Var(N) = [(b+1)(c+1)(b−a)(c−a)]/[(a+1)^2^(a+2)]* [15]. A log-transformation was used to obtain the 95% variance-based confidence intervals (CIs) of *N* so that the lower limit was always greater than the observed number of cases.

### Risk estimation

Black et al. estimated the background rate of spontaneous abortion to be 3.5 to 22.4 per 100 pregnancies, varying by age and country [16]. For the general pregnant population in Taiwan, background rates were 12.8 (95% CI, 12.8–12.9) spontaneous abortions per 100 pregnancies according to the published literature [17]. We calculated the capture-recapture estimated risks of spontaneous abortion in pregnant women who received the 2009 H1N1 vaccines. The denominator data on 2009 H1N1 vaccine doses administered to pregnant women between November 1, 2009 and August 31, 2010 were obtained from the National Influenza Vaccine Information System. The Influenza Vaccine Information System monitors 2009 H1N1 vaccine utilizations in real-time; the data distinguished doses administered by vaccine type (the unadjuvanted or MF59®-adjuvanted vaccine), but not by the timing of vaccination in relation to stage of pregnancy [9].

## Results

From November 1, 2009 through August 31, 2010, 14,474 pregnant women received the 2009 H1N1 vaccines. Of the 14,474, 13,199 (91%) received the unadjuvanted and 1,275 (9%) received the MF59®-adjuvanted vaccine. The enhanced passive surveillance received 35 AEFI reports after 2009 H1N1 vaccination in pregnant women (Table 1). We verified 37 outcomes (one woman was pregnant with triplets), including 31 pregnancy-specific adverse events (16 spontaneous abortions, 11 stillbirths, 4 neonatal deaths), 4 nonpregnancy-specific adverse events, and 2 inadvertent immunizations in vaccine recipients who were unaware of pregnancy at time of vaccination (Table 2). In 8 of the 35 reports, placental/cord pathology or fetal/infant autopsy was performed. The findings included chorioamnionitis (n = 2), trisomy 21 (n = 1), cerebral hemangioblastoma (n = 1), meconium aspiration syndrome (n = 1), stenosis of villi vessels (n = 1), cord stricture (n = 1), and placenta accreta (n = 1).

**Table 1:** Characteristics of reports following 2009 H1N1 vaccines among pregnant women, November 1, 2009–August 31, 2010, by vaccine type.

Characteristic	Vaccine type	
	Unadjuvanted vaccine	MF59®-adjuvanted vaccine
	(n = 28)	(n = 7)
Serious reports^a^, n (%)	14 (50)	3 (43)
Maternal deaths, n (%)	0 (0)	0 (0)
Median maternal age (range), y	34 (18–42)	31 (21–36)
Median onset interval (range), d	19.5 (0–70)	32 (7–46)
Median gestational age at time of onset (range), w	16.5 (6–40)	20 (15–37)
Gestational age^b^ at time of vaccination, n (%)		
First trimester	10 (36)	4 (57)
Second trimester	13 (46)	1 (14)
Third trimester	5 (18)	2 (29)

a Reports were categorized as serious if they resulted in death, life-threatening illness, hospitalization, prolongation of an existing hospitalization, permanent disability, or congenital anomaly.

b First trimester, 0–13 weeks; second trimester, 14–27 weeks; third trimester, ≥28 weeks of pregnancy.

**Table 2:** Adverse events following 2009 H1N1 vaccines among pregnant women, November 1, 2009–August 31, 2010, by vaccine type and timing of vaccination.

Adverse event	Timing of vaccination^a^					
	First trimester		Second trimester		Third trimester	
	Unadjuvanted vaccine	MF59®-adjuvanted vaccine	Unadjuvanted vaccine	MF59®-adjuvanted vaccine	Unadjuvanted vaccine	MF59®-adjuvanted vaccine
	(n = 10)	(n = 4)	(n = 15)	(n = 1)	(n = 5)	(n = 2)
Pregnancy-specific						
Spontaneous abortion	9	1	6	0	0	0
Stillbirth	0	0	7	1	2	1
Neonatal death	0	0	2^b^	0	1^c^	1^d^
Nonpregnancy-specific	1^e^	1^f^	0	0	2^f^	0
Inadvertent immunization	0	2^g^	0	0	0	0

a First trimester, 0–13 weeks; second trimester, 14–27 weeks; third trimester, ≥28 weeks of pregnancy.

b The causes of death were fetal anemia and hydrops fetalis (n = 1) and preterm delivery at 21^st^ week of gestation (n = 1).

c The cause of death was hydrops fetalis caused by α-thalassemia.

d The cause of death was cerebral hemangioblastoma with intracranial hemorrhage.

e The patient received the 2009 H1N1 vaccine at 10^th^ week of pregnancy and developed allergic vasculitis 10 days after vaccination. She was treated with systemic corticosteroids, which led to an elective termination of pregnancy due to perceived risk of corticosteroid on fetal development.

f The reported adverse events were generalized rash for the MF59®-adjuvanted vaccine; and numbness of fingers (n = 1) and dizziness, tremor, and rhinorrhea (n = 1) for the unadjuvanted vaccine.

g One of the pregnant women delivered a healthy male infant at 39^th^ week of gestation. The outcome for the other pregnant woman was not specified in the report.

### Reports after the unadjuvanted vaccine

Among 13,199 pregnant women who received the unadjuvanted 2009 H1N1 vaccine, 15 spontaneous abortions, 9 stillbirths, 3 neonatal deaths, and 3 nonpregnancy-specific adverse events were reported (Table 2). The median days from 2009 H1N1 vaccination to the occurrence of spontaneous abortion was 17 days (range, 0–45 days). In 7 of the 15 spontaneous abortion reports, advanced maternal age (≥35 years) were identified. One spontaneous abortion occurred in a woman who was 36 years of age and received the vaccine at 10^th^ week of pregnancy; cytogenetic analysis identified trisomy 21. The median days from 2009 H1N1 vaccination to the occurrence of stillbirth was 18 days (range, 1–70 days). All of the 9 stillbirths reported more than one risk conditions associated with stillbirth [18], including maternal age ≥35 years (n = 3), chorioamnionitis (n = 2), preterm premature rupture of membranes (n = 2), gestational diabetes mellitus (n = 1), hyperthyroidism (n = 1), preeclampsia (n = 1), multiple gestation (n = 1), small for gestational age fetus (n = 1), fetal hydrocephalus due to intraventricular hemorrhage (n = 1), placental insufficiency (n = 1), and oligohydramnios (n = 1). Cause of death for the three neonates varied (Table 2). No major birth defect was observed for stillborn fetuses or live-born infants who died in the neonatal period.

The three reports involving nonpregnancy-specific adverse events included allergic vasculitis (n = 1), numbness of fingers (n = 1), and dizziness, tremor, and rhinorrhea (n = 1). The allergic vasculitis patient received the unadjuvanted 2009 H1N1 vaccine at 10^th^ week of pregnancy and reported onset of adverse events 10 days after vaccination. She was treated with systemic corticosteroids, which led to an elective termination of pregnancy due to perceived risk of corticosteroids on fetal development.

### Reports after the MF59®-adjuvanted vaccine

Reports involving the 1,275 pregnant women who received the MF59®-adjuvanted 2009 H1N1 vaccine included one spontaneous abortion, two stillbirths, one neonatal death, one nonpregnancy-specific adverse event, and two inadvertent immunizations (Table 2). Chorioamnionitis was reported in the spontaneous abortion that occurred at 15^th^ week of pregnancy, 46 days after 2009 H1N1 vaccination. Onset days from 2009 H1N1 vaccination to the occurrence of stillbirth was 7 and 32 days, respectively. Maternal age was ≥35 years in one stillbirth; for the other, no relevant risk condition associated with stillbirth could be identified. For the infant who died within one month of birth, cause of death was cerebral hemangioblastoma with intracranial hemorrhage. The nonpregnancy-specific adverse event was generalized skin rash in a woman 28 years of age, 30 days after receipt of the MF59®-adjuvanted 2009 H1N1 vaccine at 11^th^ week of pregnancy.

### Capture-recapture estimators and risk assessment

Sixteen cases of spontaneous abortion after 2009 H1N1 vaccination were identified in the passive surveillance database and 135 cases were identified in the LLDB; 6 matches occurred between the two sources. We estimated that the true number of spontaneous abortion in pregnant women who received the 2009 H1N1 vaccines to be 329 (95% CI, 196–553) and reporting completeness of the passive surveillance to be 5% (95% CI, 3%–8%). The estimated risk of spontaneous abortion for the 14,474 pregnant women who received 2009 H1N1 vaccines was 2.3 (95% CI, 1.4–3.8) per 100 pregnancies, compared with a local background rate of 12.8 (95% CI, 12.8–12.9) per 100 pregnancies.

Table 3 showed the number of spontaneous abortion ascertained from the combinations of capturing data sources for different types of 2009 H1N1 vaccines. We estimated that risk of spontaneous abortion was 2.2 (95% CI, 1.3–4.0) and 2.0 (95% CI, 2.0–2.0) per 100 pregnancies associated with the unadjuvanted and MF59®-adjuvanted vaccine, respectively.

**Table 3:** Distribution of cases of spontaneous abortion following 2009 H1N1 vaccines from the passive surveillance and large-linked databases, November 1, 2009–August 31, 2010, by vaccine type.

Vaccine type	Capturing source		Number of cases ascertained	Capture-recapture estimator of total cases (95% CI^a^)
	Passive surveillance database	Large-linked database		
Unadjuvanted vaccine	Yes	Yes	5	295 (167–522)
	Yes	No	10	
	No	Yes	105	
MF59®-adjuvanted vaccine	Yes	Yes	1	25 (25–25)
	Yes	No	0	
	No	Yes	24	

a CI, confidence interval.

## Discussion

We summarized the adverse event profile of 14,474 pregnant women who received the 2009 H1N1 vaccines in Taiwan. Most adverse events reported were consistent with those described following administration of inactivated seasonal influenza vaccine to pregnant women [19]. Review of reports did not find any concerning pattern of adverse pregnant or fetal outcomes.

The inactivated seasonal influenza vaccine without adjuvant has been considered safe when administered during pregnancy [4]–[6], [20], and the few postlicensure studies that have been published also supported the safety of unadjuvanted 2009 H1N1 vaccine in pregnant women [21], [22]. Compared with the unadjuvanted product, fewer data have been available on exposure to MF59®-adjuvanted influenza vaccine in pregnancy. A review of the Novartis pregnancy database for 43 reported exposures to MF59®-adjuvanted influenza vaccines found that the distribution of pregnant outcomes was similar in subjects exposed to MF59®-adjuvanted and unadjuvanted products at any time of pregnancy [8]. However, the inclusion of only 43 pregnancies limited the ability to conclude on risks associated with exposure to MF59®-adjuvanted influenza vaccines [23]. Our study provided one of the few assessments on the safety of 2009 H1N1 vaccine adjuvanted with MF59® based on postlicensure data involving 1,275 pregnant recipients. Data are reassuring regarding the safety of MF59®-adjuvanted 2009 H1N1 vaccine in pregnancy.

AEFI reporting rates in our study needed cautious interpretation because data collected through the passive surveillance was underreported [12]. Reporting completeness of passive AEFI surveillance can vary but is not routinely available [13], [24], [25]. Information on the magnitude of underreporting would be essential to evaluate an association between 2009 H1N1 vaccine and a reported adverse event. In Taiwan, reporting completeness of the passive surveillance for spontaneous abortion after 2009 H1N1 vaccination was estimated to be 5%, but accuracy of this estimate was limited by the equal likelihood of capture assumption of the capture-recapture method [26]. Spontaneous abortion with increasing onset interval from vaccination was less likely to be captured by the passive surveillance system [13], [24]. In addition, the type of facility where a person received the 2009 H1N1 vaccine could also affect case ascertainment by the LLDB. At provider offices, vaccination details were electronically transmitted to the LLDB through NHI computerized database; however, immunization records at schools, workplaces, or mass vaccination stations were maintained in paper forms and would have to be manually computerized [9]. Pregnant women who received the 2009 H1N1 vaccine at provider offices were more likely than others vaccinated elsewhere to be captured by the LLDB.

The estimated risks of spontaneous abortion in pregnant women receiving the unadjuvanted or MF59®-adjuvanted product were lower than background rates for the general pregnant population [16], [17]. The comparisons to background rates, however, have some limitations. Most spontaneous abortions occur within the first trimester of pregnancy and the rate declines throughout pregnancy [27]. Our study did not allow risk calculations for each trimester because the denominator data was based on number of 2009 H1N1 vaccine doses administered to women at all stages of pregnancy; therefore, we may have underestimated the risk of spontaneous abortion after 2009 H1N1 vaccination. Receipt of 2009 H1N1 vaccine is voluntary and thus may be preferentially sought by motivated and healthier individuals. In practice, clinicians may not have vaccinated patients perceived to be at risk for adverse pregnant outcomes. These would result in a preferential receipt of 2009 H1N1 vaccines by a relatively healthy pregnant population (“healthy vaccinee phenomenon”) [28]. Spontaneous abortions have been reported among pregnant women with 2009 H1N1 infections [3]. There also have been reports of higher miscarriage rates during previous influenza pandemics [29], [30]. Pregnant women who received the 2009 H1N1 vaccine might have been protected from influenza infection and therefore, were less likely to develop spontaneous abortions than the unvaccinated group.

This study shared other inherent limitations of all passive surveillance systems [12]. Although clinical reviews of medical records for reports involving pregnancy-specific adverse events could improve data quality and completeness, limitations such as reporting biases and lack of a controlled unvaccinated remained. Therefore, these data cannot be used to determine whether a vaccine causes an adverse event. Our study collected only information about adverse events that occurred after the administration of 2009 H1N1 vaccine and did not have all the information essential for epidemiologic assessments of causality. It is not known if the women who received the 2009 H1N1 vaccine were different in baseline characteristics or at higher risk for adverse pregnant or fetal outcomes compared with the general pregnant population.

Nevertheless, the passive AEFI surveillance provided rapid initial assessment of adverse pregnant and fetal outcomes, but it is only part of the 2009 H1N1 vaccine safety monitoring activities in Taiwan [9]. Taiwan has developed an infrastructure to actively follow up with women who received the 2009 H1N1 vaccines at different stages of pregnancy, as well as to follow their fetal and newborn outcomes, using comparison groups [9]. Data from these ongoing studies can provide further information on the safety of 2009 H1N1 vaccines in pregnant women.

## Supporting Information

Table S1 **Evaluation of reported pregnancy complications following receipt of 2009 H1N1 vaccines.**
(DOC)
