# Supplementary material for: Adverse Events Following Pandemic A (H1N1) 2009 Monovalent Vaccines in Pregnant Women — Taiwan, November 2009–August 2010
Source: PLoS One. 2011 Aug 5;6(8):e23049. doi: 10.1371/journal.pone.0023049 (PMC3151285; doi:10.1371/journal.pone.0023049)
Supplement: Table S1 — Evaluation of reported pregnancy complications following receipt of 2009 H1N1 vaccines. (DOC) [file pone.0023049.s001.doc]

**Table S1.** Evaluation of reported pregnancy complications following receipt of 2009 H1N1 vaccines

| Complete blood count with differential  Blood ABO and Rh typing  AST, ALT, blood urea nitrogen, creatinine  Fasting glucose  TSH, free thyroxine  Lupus anticoagulant  Anticardiolipin and anti-β2 glycoprotein I antibodies  Fibrinogen  Kleihauer-Betke test for fetomaternal hemorrhage  Indirect Coombs’ test  TORCH and syphilis serology  Placental cultures  Placenta, cord blood, and amniotic fluid rRT-PCR for TORCH, influenza virus, parainfluenza virus, respiratory syncytial virus, adenovirus, rhinovirus  Placental and umbilical cord pathology  Fetal or infant autopsy  Karyotype |
| --- |

Abbreviations: AST, alanine aminotransferase; ALT, aspartate aminotransferase; TSH, thyroid-stimulating hormone; TORCH, toxoplasmosis, parvovirus, rubella, varicella-zoster virus, cytomegalovirus, Epstein-Barr virus, enterovirus, and herpes simplex virus; rRT-PCR, real-time reverse-transcriptase polymerase chain reaction
